# CRISPR Is an Optimal Target for the Design of Specific PCR Assays for *Salmonella enterica* Serotypes Typhi and Paratyphi A

**DOI:** 10.1371/journal.pntd.0002671

**Published:** 2014-01-30

**Authors:** Laetitia Fabre, Simon Le Hello, Chrystelle Roux, Sylvie Issenhuth-Jeanjean, François-Xavier Weill

**Affiliations:** Unité des Bactéries Pathogènes Entériques, Centre National de Référence des *Escherichia coli*, *Shigella* et *Salmonella*, WHO Collaborative Center for Reference and Research on *Salmonella*, Institut Pasteur, Paris, France; Massachusetts General Hospital, United States of America

## Abstract

**Background:**

Serotype-specific PCR assays targeting *Salmonella enterica* serotypes Typhi and Paratyphi A, the causal agents of typhoid and paratyphoid fevers, are required to accelerate formal diagnosis and to overcome the lack of typing sera and, in some situations, the need for culture. However, the sensitivity and specificity of such assays must be demonstrated on large collections of strains representative of the targeted serotypes and all other bacterial populations producing similar clinical symptoms.

**Methodology:**

Using a new family of repeated DNA sequences, CRISPR (clustered regularly interspaced short palindromic repeats), as a serotype-specific target, we developed a conventional multiplex PCR assay for the detection and differentiation of serotypes Typhi and Paratyphi A from cultured isolates. We also developed EvaGreen-based real-time singleplex PCR assays with the same two sets of primers.

**Principal findings:**

We achieved 100% sensitivity and specificity for each protocol after validation of the assays on 188 serotype Typhi and 74 serotype Paratyphi A strains from diverse genetic groups, geographic origins and time periods and on 70 strains of bacteria frequently encountered in bloodstream infections, including 29 other *Salmonella* serotypes and 42 strains from 38 other bacterial species.

**Conclusions:**

The performance and convenience of our serotype-specific PCR assays should facilitate the rapid and accurate identification of these two major serotypes in a large range of clinical and public health laboratories with access to PCR technology. These assays were developed for use with DNA from cultured isolates, but with modifications to the assay, the CRISPR targets could be used in the development of assays for use with clinical and other samples.

## Author Summary

Typhoid and paratyphoid fevers are a major health problem in developing countries, where they are still endemic. Accurate diagnosis remains a challenge, as identification of the causal agents, *Salmonella enterica* serotypes Typhi and Paratyphi A, respectively, requires appropriate facilities (for bacterial culture) and specific reagents (typing antisera). Several *Salmonella* serotype-specific PCR assays have been published and even used directly on biological samples, but none has been validated on a large collection of strains to check for reliability and robustness. We report here the successful development of conventional and real-time PCR assays for the identification of serotypes Typhi and Paratyphi A from cultured isolates. These assays, based on an original target (CRISPR), had a sensitivity and specificity of 100% in tests on a collection of more than 330 strains. The conventional multiplex PCR assay provides an alternative to the serotyping method for laboratories in developing countries unable to obtain a regular supply of typing antisera but with access to PCR technology.

## Introduction

Typhoid and paratyphoid fevers remain a major health problem in the developing world, particularly in South East Asia and Africa, with estimates of more than 20 million illnesses and over 200,000 deaths due to typhoid fever and more than 5 million illnesses due to paratyphoid fever in 2000 [1]. The causal agents of these diseases, *Salmonella enterica* serotypes Typhi and Paratyphi A, respectively, must be identified early and accurately, to ensure appropriate antimicrobial therapy for the individual concerned, particularly in endemic areas in which the main differential diagnosis is malaria. Correct identification is also fundamental for prevalence and monitoring studies of typhoid and paratyphoid fevers, for the implementation, strengthening and evaluation of health policies (vaccination campaigns, sanitation, water supply treatment, empirical antimicrobial therapy). In high-income countries, in which typhoid fever mostly affects travelers, laboratory confirmation is based on culture, mostly semi-automated blood culture, and further serotyping and antimicrobial drug susceptibility testing of the bacterial isolates. The cost of typing sera and the generalization of accreditation for private laboratories have resulted in the restriction of serotyping to public health laboratories or national reference centers, delaying diagnosis by several days. Furthermore, some isolates of serotypes Typhi and Paratyphi A may not be serotypable due to rough phenotypes (autoagglutination due to the complete or partial loss of lipopolysaccharide) and non motility, and some isolates of serotype Typhi may be classified as not serotypable if overexpression of the Vi polysaccharide masks the O antigens and the Vi antigen is not destroyed by heating before O agglutination. In the regions in which these diseases are endemic, both culture and serotyping are considered expensive and are rarely used. Thus, despite their low sensitivity and/or specificity, serological tests, such as the Widal test, are the predominant method for typhoid diagnosis in these regions [2].

Molecular PCR-based methods have been developed [3]–[14] (Table 1), to overcome these problems of the need for blood culture (requirement for specialist microbiological facilities, low sensitivity). Given the small numbers of circulating bacteria (<1/ml during infection), several strategies have been applied, involving a broth pre-enrichment step [11], [12], nested PCR [3] or real-time PCR [13]. Various molecular targets have been used (Table 1). Initially, the targets chosen were based on genetic structures encoding somatic (O) and/or flagellar (H) antigens and/or Vi antigen [3]–[5], [10]–[12]. More recently, *in silico* comparative genomics has been used to identify serotype-specific regions [6]–[9], [13], [14].

**Table 1:** Published PCR assays targeting *S. enterica* serotypes Typhi and/or Paratyphi A.

Targeted serotypes	Target genes	Target selection by comparative genomics	Type of PCR assay	Bacterial strains tested (no./origin)	Clinical samples tested (no./origin)	Detection limits^d^	Ref.
Typhi	*fliC*-d‡	No	Nested	Typhi (2), other *Salmonella* serotypes (8), non–*Salmonella* (9)	Whole blood (12/Korea)	10 cfu/ml	3
Typhi, Paratyphi A	*tyv* *, *prt* *, *viaB* §, *fliC*-d‡, *fliC*-a‡	No	Conventional	Typhi (15/Japan), Paratyphi A (11/Japan), other *Salmonella* serotypes (19), non–*Salmonella* (26)	No	NT	4
Typhi†, Paratyphi A	*rfbJ* *, *tyv* *, *prt* *, *viaB* §, *fliC*-d‡, *fliC*-a‡	No	Conventional	Typhi (136/Mali, Chile), Paratyphi A (33/Chile), other *Salmonella* serotypes (541)	No	NT	5
Paratyphi A	spa0180 (*stkF*)^a^, spa2473^a^, spa2539^a^, spa4289 (*hsdM*)^a^	Yes	Conventional	Paratyphi A (52/Malaysia), other *Salmonella* serotypes (75), non–*Salmonella* (14)	No	1×10^5^ cfu/ml	6, 7
Typhi††	STY1599^b^	Yes	Conventional	Typhi (31/Korea), other *Salmonella* serotypes (43), non–*Salmonella* (37)	No	NT	8
Typhi, Paratyphi A	STY0312^b^ ( = SPA2476^c^), STY0313–STY0316^b^	Yes	Conventional	Typhi and Paratyphi A (35, India), other *Salmonella* serotypes (14), non–*Salmonella* (11)	Whole blood (78/India)	4 cfu/ml	9
Typhi	*tyv* *, *viaB* §, *ratA*, *fliC*-d‡,	No	Conventional	Typhi (1), other *Salmonella* serotypes (1)	Sera (18, India)	5 fg	10
Typhi	*fliC*-d‡	No	Conventional	Typhi (1)	No	3×10^5^ cfu/ml	11, 12
Typhi, Paratyphi A	STY0201^b^, SSPA2308^c^	Yes	Real-time	Typhi (81/Nepal), Paratyphi A (61/Nepal), other *Salmonella* serotypes (10), non–*Salmonella* (13)	Whole blood (100/Nepal), bone marrow (28/Vietnam)	2.5×10^2^ cfu/ml (Typhi)	13
Typhi†††, Paratyphi A	*stg*A, SPA1723a-SSPA1724^a^ intergenic region, STY4220^b^	Yes	Conventional	Typhi (14/Singapore), Paratyphi A (7/Singapore), other *Salmonella* serotypes (103), non–*Salmonella* (8)	No	1×10^5^ cfu/ml (Typhi), 2×10^5^ cfu/ml (Paratyphi A)	14
Typhi, Paratyphi A	CRISPR	Yes	Conventional and real-time	Typhi (188/global), Paratyphi A (74/global), other *Salmonella* serotypes (29), non–*Salmonella* (42)	No	1.7 pg (Typhi), 3 pg (Paratyphi A)	This study

This multiplex assay also aims to detect serotype Paratyphi B,

This multiplex assay also aims to detect serotypes Typhimurium, Enteritidis, *S. enterica* subsp. *enterica* and *Salmonella* spp.,

This multiplex assay also aims to detect *Salmonella* spp.,

O antigen,

Vi antigen,

H antigen,

^a^ from the serotype Paratyphi A ATCC 9150 genome,

^b^ from the serotype Typhi CT18 genome,

^c^ from the serotype Paratyphi A AKU_12601 genome.

^d^ Results for detection limits are presented in colony-forming units (cfu)/ml when determined with artificially inoculated blood samples (without broth enrichment) or in amount of DNA per PCR when determined by 10-fold dilutions of the bacterial culture; NT, not tested.

**Figure 1:**
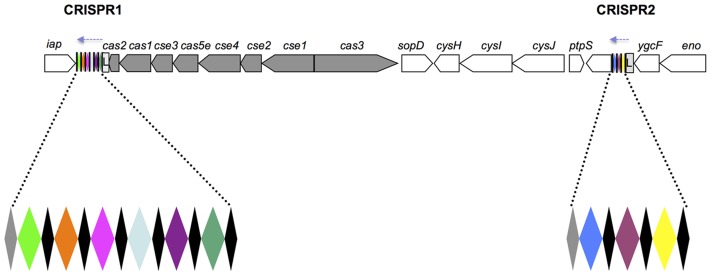
Structure of the CRISPR/Cas system from *S. enterica* serotypes Typhi and Paratyphi A. Two CRISPR loci (CRISPR1 and CRISPR2) flank the CRISPR-associated sequences (cas). The *cas* genes, spacers, direct repeats, leader sequences are represented by gray arrows, colored diamonds, black diamonds, and light gray boxes marked L, respectively. The genomic orientation from *iap* to *ygcF* has been maintained to ensure consistency with previous studies [15], [28], [29]. The probable transcriptional orientation of the CRISPR loci, as extrapolated from studies in *E. coli* [30], [31], is indicated by light blue dashed arrows.

We recently described a new molecular method for *Salmonella* typing, based on polymorphism of the CRISPR (clustered regularly interspaced short palindromic repeats) region [15]. This region contains a new family of repeated DNA sequences [16] characterized by 24–47 bp (29 bp for *Salmonella*) direct repeats separated by variable 21–72 bp (32 bp for *Salmonella*) sequences known as “spacers”. In *Salmonella*, this region consists of two CRISPR loci (CRISPR1 and CRISPR2) flanking the Cas machinery (Figure 1). The analysis of 783 strains and genomes from 130 serotypes, including 18 strains and two genomes of serotype Typhi and 12 strains and two genomes of serotype Paratyphi A, led to the identification of more than 3,800 different spacers and showed that the spacer content of a strain was strongly correlated with its serotype. Furthermore, the presence of unique, constant spacers in certain serotypes, such as Typhi and Paratyphi A, was thought to constitute an optimal molecular target for the development of serotype-specific PCR assays. This approach has since been used by Delannoy *et al.* [17], [18] for the identification of eight major serotypes of enterohemorrhagic *Escherichia coli*.

The aim of this work was to provide proof-of-principle that CRISPR polymorphism can be used for the development of specific PCRs targeting serotypes of interest. We report here the successful development and validation of singleplex and multiplex, conventional and real-time PCRs for the rapid identification of serotypes Typhi and Paratyphi A from cultured isolates.

## Methods

### *In silico* analysis

We used CRISPR sequencing data from a previous study, which included 18 strains and two genomes (Ty2 and CT18, GenBank accession nos. AE014613 and AL627276, respectively) of *S. enterica* serotype Typhi and 12 strains and two genomes (ATCC 9150 and AKU_12601, GenBank accession nos. CP000026 and FM200053, respectively) of *S. enterica* serotype Paratyphi A [15]. A new genome (P-stx-12, GenBank accession no. CP003278) of serotype Typhi was also analyzed.

### Bacterial strains and isolates

For assessment of the sensitivity of the serotype-specific PCRs, the *S. enterica* serotype Typhi and Paratyphi A strains and isolates used in this study were selected so as to be representative of the broadest possible genetic diversity for these pathogens. We used collection strains (including the 18 strains of serotype Typhi and 12 of serotype Paratyphi A for which the CRISPR regions had been sequenced) previously shown to be representative of the principal genetic lineages of the two serotypes, together with recent clinical isolates. For serotype Typhi, we used a total of 188 strains and isolates: 79 collection strains (isolated between 1918 and 2006), including the reference strain Ty2, representative of 65 different haplotypes [19] and 109 isolates recovered in 2009. For serotype Paratyphi A, we used a total of 74 strains and isolates: 32 collection strains (isolated between 1926 and 2006), including the reference strain 1K, part of an ongoing whole-genome sequencing project, and 42 isolates recovered in 2009. All the serotype Typhi and Paratyphi A strains and isolates were from the French National Reference Center for *Escherichia coli*-*Shigella*-*Salmonella* (FNRC-ESS, Institut Pasteur, Paris) or the WHO Collaborative Center for Reference and Research on *Salmonella* (WHO- Salm, Institut Pasteur, Paris) (Table S1).

For assessment of the specificity of the two serotype-specific PCRs, we used 70 strains of bacteria, some of which are frequently encountered in bloodstream infections. These included 29 non typhoidal *Salmonella* strains (NTS) and 42 strains from 37 species from other genera. These strains were from the FNRC-ESS, the WHO-Salm, the *Collection de l'Institut Pasteur* (CIP, Institut Pasteur, Paris) or the French National Reference Center for *Neisseria* (Institut Pasteur, Paris) (Table 2).

**Table 2:** Results of conventional PCR assays targeting *S. enterica* serotypes Typhi and Paratyphi A.

*S. enterica* subsp. *enterica* serotype	No. of strains tested^1^	PCR Typhi^2^	PCR Paratyphi A^2^
Typhi	188	+	−
Paratyphi A	74	−	+
Typhimurium	1	−	−
Enteritidis	1	−	−
Dublin	1	−	−
Newport	1	−	−
Paratyphi B biovar Java	1	−	−
Paratyphi B	1	−	−
Paratyphi C	1	−	−
Choleraesuis *sensu stricto*	1	−	−
Choleraesuis var. Decatur	1	−	−
Muenchen	1	−	−
Manhattan	1	−	−
Mikawasima	1	−	−
Nitra	1	−	−
Blockley	1	−	−
Blegdam	1	−	−
Mbandaka	1	−	−
Canada	1	−	−
Indiana	1	−	−
Emek	1	−	−
Urbana	1	−	−
Muenster	1	−	−
Virchow	1	−	−
Brandenburg	1	−	−
Panama	1	−	−
Schwarzengrund	1	−	−
Poona	1	−	−
Heidelberg	1	−	−
Kentucky	1	−	−
Oranienburg	1	−	−
**Other species**			
*Escherichia coli*	5	−	−
*Escherichia blattae*	1	−	−
*Escherichia vulneris*	1	−	−
*Shigella dysenteriae* 1	1	−	−
*Shigella boydii* 1	1	−	−
*Shigella dysenteriae* 2	1	−	−
*Shigella sonnei g*	1	−	−
*Shigella flexneri* 2a	1	−	−
*Enterobacter aerogenes*	1	−	−
*Enterobacter kobei*	1	−	−
*Enterobacter gergoviae*	1	−	−
*Enterobacter cloacae*	1	−	−
*Citrobacter farmerii*	1	−	−
*Citrobacter rodentium*	1	−	−
*Citrobacter murliniae*	1	−	−
*Citrobacter werkmanii*	1	−	−
*Citrobacter sedlakii*	1	−	−
*Citrobacter braakii*	1	−	−
*Citrobacter amalonaticus*	1	−	−
*Citrobacter diversus*	1	−	−
*Edwardsiella tarda*	1	−	−
*Kluyvera cryocrescens*	1	−	−
*Pantoea agglomerans*	1	−	−
*Serratia marcescens*	1	−	−
*Leminorella grimontii*	1	−	−
*Buttauxiella agrestis*	1	−	−
*Yersinia pseudotuberculosis*	1	−	
*Providencia rettgeri*	1	−	−
*Dickeya chrysanthemi*	1	−	−
*Klebsiella pneumoniae*	1	−	−
*Morganella morganii*	1	−	−
*Proteus vulgaris*	1	−	−
*Streptococcus pyogenes*	1	−	−
*Staphylococcus aureus*	1	−	−
*Staphylococcus epidermidis*	1	−	−
*Streptococcus agalactiae*	1	−	−
*Streptococcus pneumoniae*	1	−	−
*Neisseria meningitidis*	1	−	−

^1^ The details of the strains are provided in Supplemental Table 1.

^2^+, amplicon of the expected size; −, no amplicon of the expected size.

### Genomic DNA extraction

Total DNA was extracted from reference strains with the Wizard Genomic DNA purification Kit (Promega, Wisconsin, United States). For clinical isolates, we increased the throughput of the extraction step with the InstaGene matrix kit (Bio-Rad, Marnes-la-Coquette, France). Both DNA extraction methods were performed according to the manufacturer's instructions. For PCR, we used DNA resuspended in a rehydration buffer provided with the Wizard kit or the final supernatant of the InstaGene matrix extraction process.

### Serotype-specific PCRs

The PCR specific for *S. enterica* serotype Typhi was carried out with forward primer TY-F (5′-ACGTAGACTCATCCTCGACC-3′ corresponding to region 2928811–2928830 of *S. enterica* serotype Typhi strain Ty2; GenBank accession no. NC_004631), binding 206 bp upstream from CRISPR2, and the reverse primer TY-R (5′-GCGTGTAGCAGTATTCACCA-3′ corresponding to region 2929055–2929036 of strain Ty2), binding to the DR-EntB0var1 spacer junction of CRISPR2.

The PCR specific for *S. enterica* serotype Paratyphi A was carried out with forward primer PA-F (5′-ACGGGTTGCTGTAATGATGC-3′ corresponding to region 2889276–2889295 of *S. enterica* serotype Paratyphi A strain ATCC 9150; GenBank accession no. NC_006511), binding 293 bp upstream from CRISPR1, and the reverse primer PA-R (5′-GCATCATCGCGCATAGTGTC-3′ corresponding to region 2889685–2889666 of strain ATCC 9150), binding to the ParA2 spacer of CRISPR1.

The primers were synthesized by MWG-Biotech (Ebersberg, Germany). The TY-R primer was purified by denaturing polyacrylamide gel electrophoresis (HYPUR grade), whereas the remaining primers were purified by either high-pressure liquid chromatography (HPLC grade) or reverse-phase chromatography (HPSF grade).

Singleplex PCR was performed in a reaction volume of 50 µl containing DNA (2 µl for the InstaGene matrix or 1 µl diluted 10-fold for Wizard), primers (TY-F and TY-R or PA-F and PA-R, 10 pmol each), GoTaq Flexi DNA polymerase (0.85 U) and its buffer (Promega), deoxyribonucleotide triphosphate (0.1 mM each), MgCl_2_ (1.5 mM), dimethyl sulfoxide (5%), and sterile water (Aqua B. Braun, Melsungen, Germany). The cycling conditions were as follows: 2 min of denaturation at 94°C, followed by 25 cycles of denaturation for 15 s at 94°C, annealing for 15 s at 56°C and polymerization for 30 s at 72°C and a final extension phase at 72°C for 5 min. Amplification products were visualized by electrophoresis in a 2.2% agarose gel. The sizes of the expected PCR products for serotypes Typhi (TY-F and TY-R primers) and Paratyphi A (PA-F and PA-R) strains were 244 bp and 409 bp, respectively.

Multiplex PCR was performed in a reaction volume of 50 µl containing DNA (2 µl for the InstaGene matrix or 1 µl diluted 10-fold for Wizard), TY-F and TY-R primers (10 pmol each), PA-F and PA-R (5 pmol each), GoTaq Flexi DNA polymerase (0.85 U) and its buffer (Promega), deoxyribonucleotide triphosphate (0.1 mM each), MgCl_2_ (1.5 mM), dimethyl sulfoxide (5%), and sterile water (Aqua B. Braun). The cycling conditions were as follows: 2 min of denaturation at 94°C, followed by 25 cycles of denaturation for 15 s at 94°C, annealing for 15 s at 56°C and polymerization for 30 s at 72°C and a final phase of extension, for 5 min at 72°C. Amplification products were visualized by electrophoresis in a 2.2% agarose gel.

The DNA of the reference strains of serotypes Typhi (Ty2) and Paratyphi A (1K) was used as a positive control for all these PCRs. Negative controls were obtained by replacing the DNA with sterile water (Aqua B. Braun).

### EvaGreen-based real-time PCR assay

The same primers as for standard PCR were used in singleplex reactions. For each set of primers, the optimized EvaGreen protocol was carried out in a final volume of 20 µl containing DNA (2 µl diluted 10-fold for the InstaGene matrix or 2 µl diluted 100-fold for Wizard), 10 µl of SsoFast™ EvaGreen Supermix (Bio-Rad), 100 nM PA-F and PA-R primers or 300 nM TY-F and TY-R primers and sterile water (Aqua B. Braun). PCR was carried out on a CFX96™ Real-Time PCR detection system (Bio-Rad) with the following cycling conditions: 98°C for 2 min, followed by 40 cycles of 98°C for 5 s, 62°C for 2 s and 72°C for 10 s). A melting curve analysis was performed for the amplicons obtained, immediately after the completion of PCR cycling, with a ramp of 0.5°C s^−1^ over the interval 60 to 95°C. We calculated Ct values from automatic baseline settings and the melting curve analyses were performed with CFX Manager version 2.1 (Bio-Rad).

The detection limits were determined by two methodologies. First, we amplified, in duplicate, serial dilutions of genomic Wizard DNA from the reference strains Ty2 and 1K of serotypes Typhi and Paratyphi A, respectively. The concentrations of the Wizard DNA extracts were determined with both a NanoDrop 2000 and a NanoDrop 8000 spectrophotometer (Thermo Scientific, France) and these extracts were then diluted in sterile water (10^−1^, 10^−2^, 10^−3^, 10^−4^, 10^−5^, 2×10^−5^, 4×10^−5^, 6×10^−5^, 8×10^−5^, 10^−6^ dilutions tested). Second, serial dilutions of cultures of *S. enterica* serotype Typhi Ty2 and Paratyphi A 1K cells were used as a template for PCR. An overnight culture with aeration at 37°C in trypticase soy broth (Bio-Rad) was adjusted to McFarland 0.5 then serially diluted 10-fold (from 10^−1^ to 10^−7^) with sterile water. We took a 1 ml aliquot of each dilution, in duplicate, centrifuged it for 5 min at 13.800×*g* then resuspended it in 200 µl of InstaGene Matrix (Bio-Rad). For each DNA extract, a 2 µl undiluted aliquot from InstaGene matrix and one aliquot diluted 10-fold were used as templates for PCR. Viable cell counts were obtained by plating 10 or 100 µl of each dilution of bacterial culture in duplicate on trypticase soy agar plates and incubating overnight at 37°C. DNA samples were considered positive if the Ct values were <40 and there was a clear melting curve peak at the expected temperature.

The sensitivity of each real-time PCR assay was assessed by testing either 46 isolates of serotype Typhi or 37 isolates of serotype Paratyphi A. Specificity was assessed by testing negative controls, including the 43 serotype Typhi isolates (for the Paratyphi A-specific real-time PCR assay), 37 serotype Paratyphi A isolates (for the Typhi-specific real-time PCR assay), 29 other NTS strains, 18 non-*Salmonella* strains, and sterile water (Aqua B. Braun). A 10^−2^ dilution of the Wizard DNA extracts of the Ty2 and 1K reference strains was used as a positive control for the tests.

### Statistical analysis

The mean and standard deviation of the Ct values were calculated with Excel (Microsoft).

## Results

### Design of CRISPR-based primers for serotype Typhi- and Paratyphi A-specific PCRs

**Figure 2:**
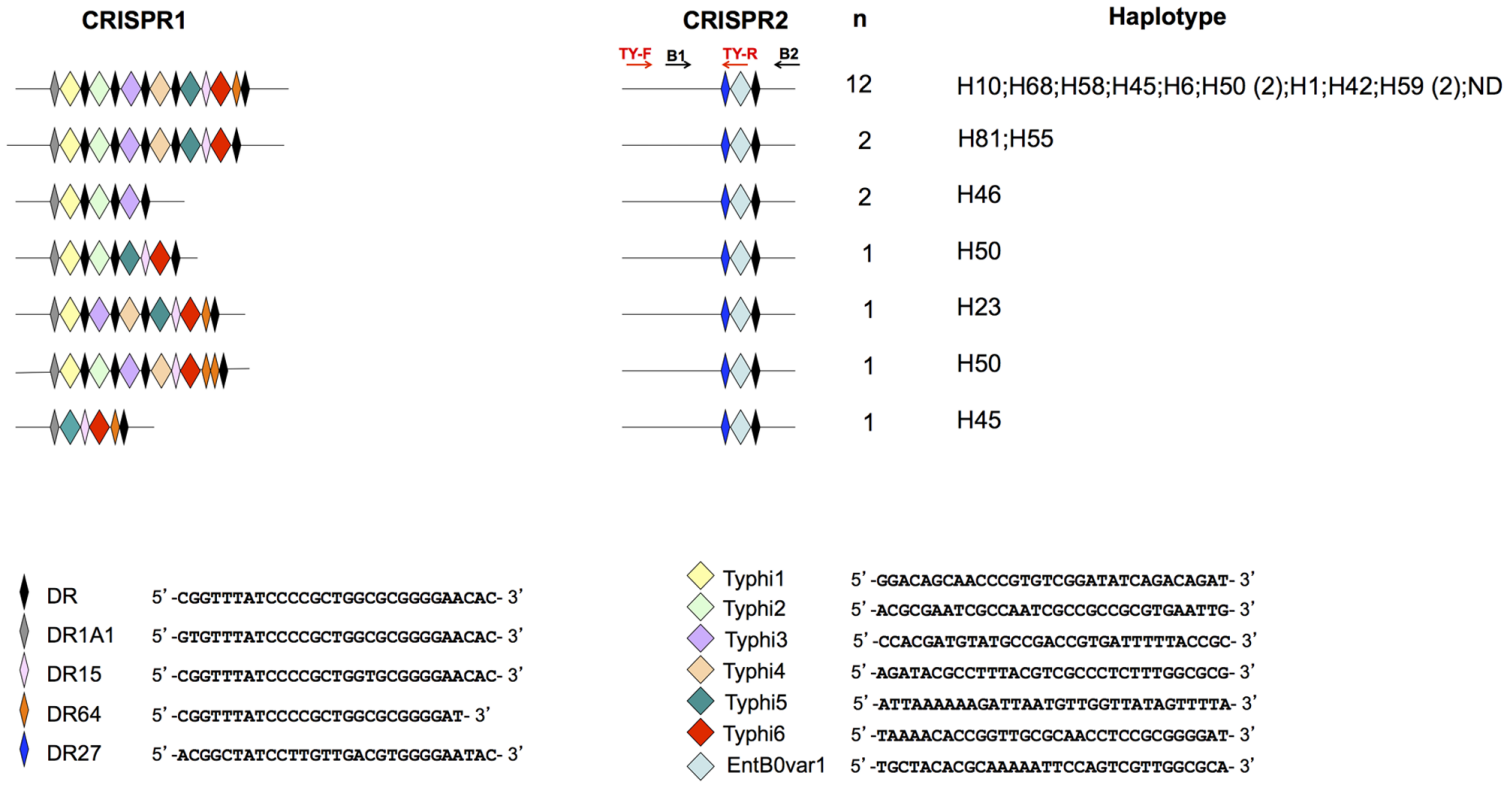
CRISPR profiles obtained for the 18 strains and two genomes of *S. enterica* serotype Typhi. The spacer sequences and direct repeat (DR) sequences are indicated. The set of strains and genomes has been reported elsewhere [15]. “n” is the number of strains harboring each profile. Haplotypes [19] are indicated, to illustrate the genetic diversity of the strains studied. ND, not determined. The primers used for serotype Typhi-specific amplification (TY-F and TY-R) and those for amplification of the entire CRISPR2 sequence (B1 and B2) [15] are indicated in different colors.

**Figure 3:**
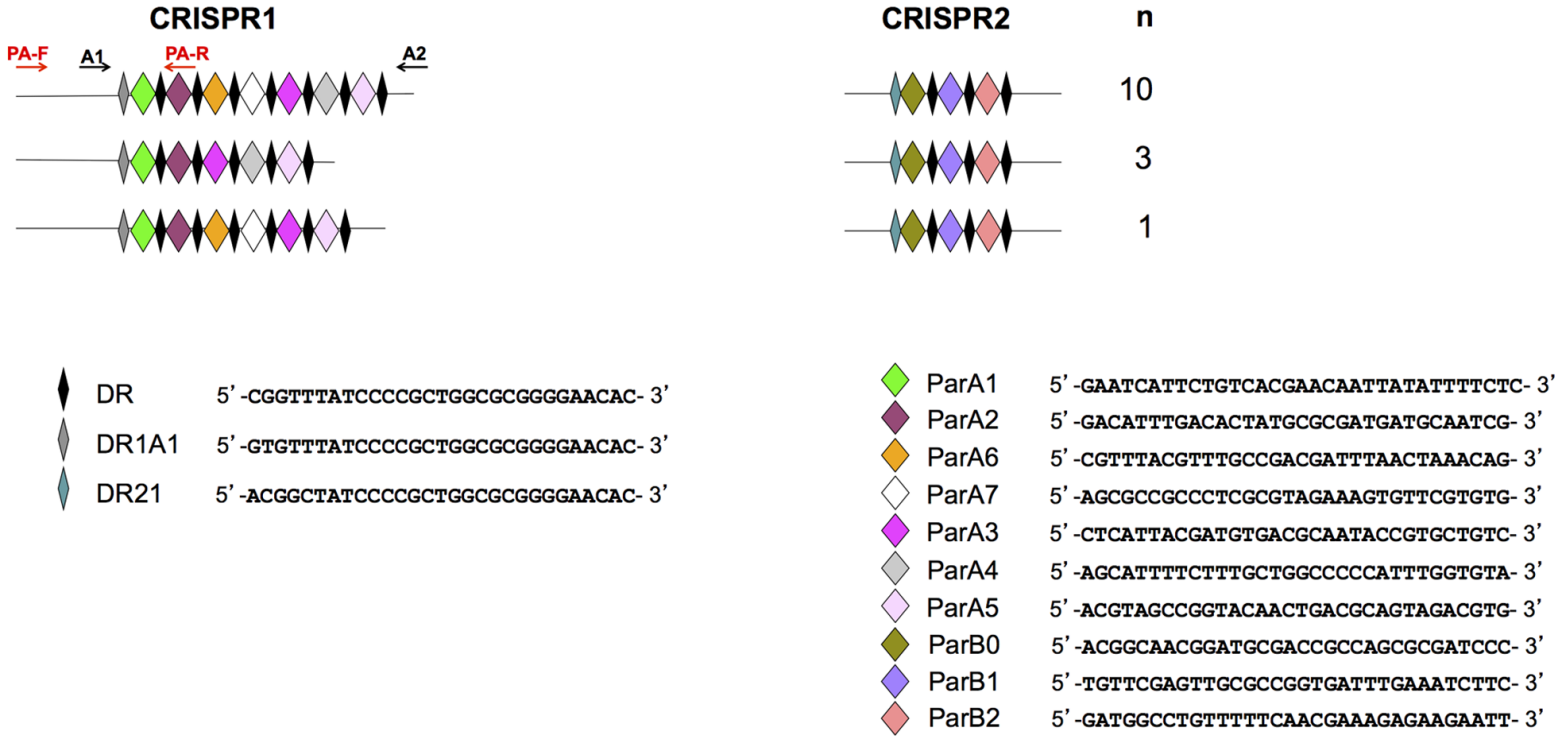
CRISPR profiles obtained for 12 strains and two genomes of *S. enterica* serotype Paratyphi A. The spacer sequences and direct repeat (DR) sequences are indicated. The set of strains and genomes has been reported elsewhere [15]. n” is the number of strains harboring each profile. The primers used for serotype Paratyphi A-specific amplification (PA-F and PA-R) and for amplification of the entire CRISPR1 sequence (A1 and A2) [15] are indicated in different colors.

The selection of targets for serotype Typhi- and Paratyphi A-specific PCRs was based on the analysis of CRISPR region polymorphism in 18 strains and two genomes (Ty2 and CT18) of serotype Typhi and 12 strains and two genomes (ATCC 9150 and AKU_12601) of serotype Paratyphi A, as previously reported [15]. Seven combined CRISPR1-CRISPR2 profiles were identified for serotype Typhi (Figure 2), and three were identified for serotype Paratyphi A (Figure 3).

**Figure 4:**
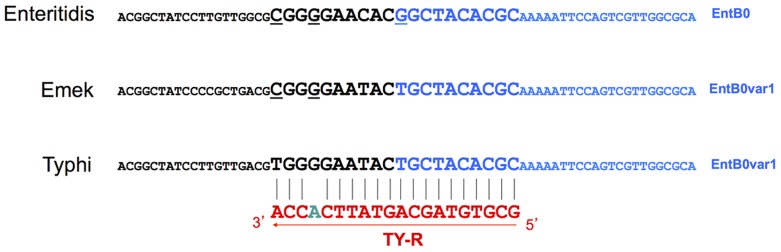
Strategy used to design TY-R, the reverse primer for the serotype Typhi-specific PCR assay. The DR sequence upstream from EntB0/EntB0var1 is indicated in black letters. The spacers EntB0 and EntB0var1 are indicated in blue letters. The nucleotides belonging to the template for primer TY-R are represented by letters of larger size. The sequence of TY-R is indicated in red, with the deliberate mismatch in green. Mismatch positions between TY-R and the templates of serotypes Emek (EntB0) and Enteritidis (EntB0var1) are underlined.

We selected CRISPR2 as the target for serotype Typhi-specific PCR, as no CRISPR1 spacer was common to all 20 strains studied or to all the genomes of this serotype. The CRISPR2 of serotype Typhi contains a unique, constant spacer, EntB0var1. However, this spacer is also known to be present as the first spacer (probably corresponding to the last transcribed) of CRISPR2 in a rare serotype, Emek [15]. It is also a single nucleotide polymorphism (SNP) variant of the EntB0 spacer, located in the first position of CRISPR2 in at least 20 serotypes, including the prevalent serotype Enteritidis [15]. The forward primer (TY-F) was designed to bind 206 bp upstream from the CRISPR2 locus (TY-F), whereas the reverse primer (TY-R) was designed to bind at the junction of EntB0var1 and its upstream DR (Figures 3 and 4), to prevent amplification in serotype Emek, which also contains EntB0var1, and in serotypes containing EntB0. The DR upstream from EntB0var1 in serotype Typhi (DR27) contains a SNP with respect to the sequences of Emek (DR1G) and Enteritidis (DR1C), and this SNP was used for annealing to the 3′ end of the HPLC-purified TY-R primer. We also deliberately incorporated a destabilizing mismatch at position −4 from the 3′-end of TY-R, to increase the specificity of the PCR [20].

For *S. enterica* serotype Paratyphi A detection, the forward primer (PA-F) was designed to bind 293 bp upstream from the CRISPR1 locus, whereas the ParA2 spacer of CRISPR1 was used as a template for the reverse primer (PA-R), as it was encountered in all the Paratyphi A strains and genomes studied and was found only in this serotype.

### PCR identification of *S. enterica* serotypes Typhi and Paratyphi A from cultured isolates

The PCR described here was developed for DNAs obtained from bacterial isolates. This PCR is based on the use of one pair of primers targeting serotype Typhi (TY-F and TY-R) and one pair of primers targeting serotype Paratyphi A (PA-F and PA-R). These primers can be used in singleplex or multiplex PCR assays.

**Figure 5:**
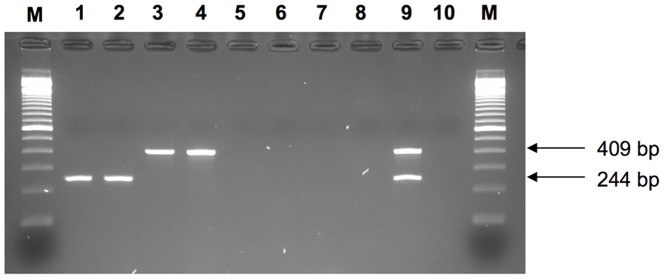
Gel electrophoresis image of the multiplex PCR used for the screening of *S. enterica* serotypes Typhi and Paratyphi A. M: 100-bp DNA ladder (Invitrogen, Carlsbad, CA, USA); lane 1, *S. enterica* serotype Typhi isolate 09-2213, lane 2, *S. enterica* serotype Typhi isolate 09-6791; lane 3, *S. enterica* serotype Paratyphi A isolate 09-2344; lane 4, *S. enterica* serotype Paratyphi A isolate 09-9426; lane 5, *S. enterica* serotype Typhimurium strain LT2; lane 6: *S. enterica* serotype Emek strain 297K; lane 7, *Escherichia coli* isolate 10-7043; lane 8: *Shigella flexneri* isolate 11-1445; lane 9, *S. enterica* serotypes Typhi and Paratyphi A reference strains Ty2 and 1K, respectively; lane 10, negative control (sterile water). We checked that the template DNA was of sufficiently high quality, by amplifying the housekeeping genes *aroC* for *Salmonella* and *adk* for *E. coli*/*Shigella* (data not shown), as previously described [32], [33].

All of the 188 strains of serotype Typhi tested yielded only the 244 bp amplicon expected for this serotype, whereas the 74 strains of serotype Paratyphi A yielded only the 409 bp amplicon expected for this serotype (Figure 5). All NTS tested gave negative results, as did the other bacterial species tested (Table 2). Similar results were obtained in singleplex and multiplex assays.

### EvaGreen-based real-time PCR assay

Each pair of primers (TY-F and TY-R; PA-F and PA-R) was tested separately on a subset of DNAs, in duplicate.

**Figure 6:**
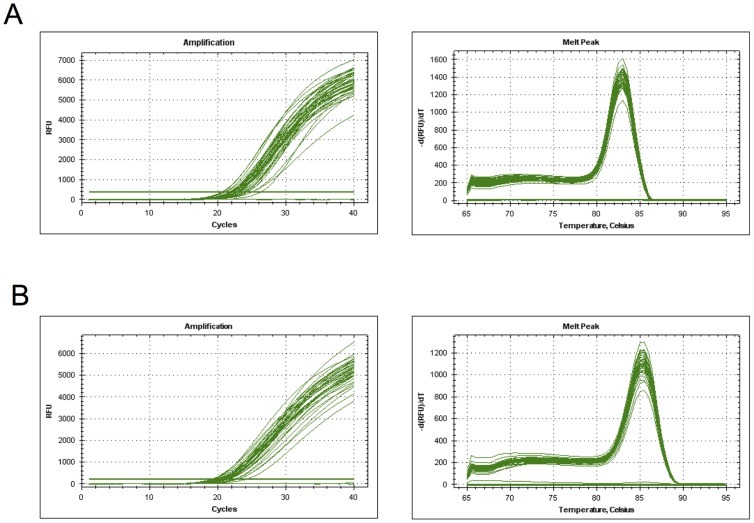
EvaGreen real-time PCR assay. (A) Amplification and melting curves for 43 serotype Typhi isolates tested in duplicate with the TY-F and TY-R primers, yielding a mean Ct value of 22.6±1.1 and a single melting curve peak at 82.5–83°C. The two negative controls (*S. enterica* serotype Paratyphi A 1K and sterile water), also tested in duplicate, appear as flat lines. (B) Amplification and melting curves for 37 serotype Paratyphi A isolates tested in duplicate with the PA-F and PA-R primers, yielding a mean Ct value of 23.1±1.1 and a melting curve peak at 85–85.5°C. The two negative controls (*S. enterica* serotype Typhi Ty2 and sterile water), also tested in duplicate, appear as flat lines.

In the serotype Typhi-specific real-time PCR assay, all 45 DNAs from the serotype Typhi isolates randomly selected from those isolated at the FNRC-ESS in 2009, together with the reference strain Ty2, gave Ct values between 20.5 and 26.2 (mean value 22.8±1.7) and a single melting curve peak at 82.5–83°C in fluorescence melting curve analysis. No positive results were obtained for the 37 serotype Paratyphi A strains, the 29 NTS strains (including serotype Emek, Dublin and Enteritidis strains) or the other bacterial species (18 tested) (Figure 6). Only one strain (of serotype Emek) had a Ct value below 40 (38.6) but no melting curve peak.

In the serotype Paratyphi A-specific assay, all 36 DNAs from the serotype Paratyphi A isolates randomly selected from those isolated at the FNRC-ESS in 2009, together with the reference strain 1K, gave Ct values between 21.2 and 26.5 (mean value 23.1±1.1) and a single melting curve peak at 85–85.5°C. No positive results (Ct values always >40 and no melting curve peak) were obtained for the 43 serotype Typhi strains, the other NTS strains or the other bacterial species (Figure 6).

The limit of detection of the EvaGreen-based real-time PCR assay was 1.7 pg for Typhi and 3 pg for Paratyphi A, for the testing of series of purified DNA extract dilutions for the reference strain of each serotype. A sensitivity of ≈60 viable bacterial cells per PCR for Typhi and ≈90 for Paratyphi A was obtained when serial dilutions of bacterial cell cultures were used as the PCR template.

## Discussion

We show here that CRISPR regions could be used as an original target for the development of PCR assays specific for particular *Salmonella* serotypes containing constant and unique spacers. As a proof-of-principle, we have successfully developed such PCRs for serotypes Typhi and Paratyphi A, the causal agents of typhoid and paratyphoid fevers.

Traditionally, the identification of these two serotypes has been based on serotyping, which requires the culture of the isolate and an adequate set of polyclonal rabbit antisera. However, these antisera are costly and are therefore not always available for use in the clinical or public health laboratories of the regions in which these diseases are endemic. Furthermore, some isolates cannot be serotyped, due to changes in the structure of the lipopolysaccharide or its accessibility to typing O antisera, or to the synthesis or assembly of the flagellum structure. PCR is increasingly being used, but the main challenge in such approaches is the identification of a stable molecular target specific to the serotype concerned. The molecular targets initially selected were related to serotype: genes involved in the biosynthesis of the flagellar (*fliC* and *fljB*) and/or O-polysaccharide (encoded by the *rfb* locus) Vi antigens. However, none of these genes, in isolation, can be considered specific for the identification of serotypes Typhi and Paratyphi A. Indeed, the Vi locus is not always present in serotype Typhi [21], [22], and *fliC-d* is not sufficient to affirm an identification of serotype Typhi, as it is also common to 145 NTS serotypes [23]. Thus, for 100% specificity to be achieved with this approach, several targets must be incorporated into the PCR assay. A new approach for identifying targets on the basis of genome comparison analysis has recently been described. However, the complete *Salmonella* genomes available cannot really be considered representative of the entire genus (39 complete genomes online, covering only 25 serotypes from the >2,500 described when this work was begun in 2011). Moreover, only three assembled genomes of serotypes Typhi (Ty2, CT18, and P-stx-12) and two of Paratyphi A (ATCC 9150 and AKU_1260) are available online. Seventeen 454 shotgun-sequenced serotype Typhi strains representing various genetic lineages [24] have been deposited in whole-genome sequence databases since 2008, but have rarely been used for the selection of serotype-specific targets [13]. This ideal approach for identifying good targets may therefore currently be biased by the limited number of genome sequences available. Furthermore, the CRISPR loci comprise only arrays of DR-spacer units in non coding regions, and this interesting target has therefore generally been ignored in comparative genomics studies, in which targets have generally been selected from open reading frames (ORFs).

Another key limitation of many published studies on serotype-specific PCR assays is the application of the validation step to a limited number of strains and/or blood samples, collected locally (Table 1). In our study, a sensitivity of 100% (i.e., the stability of the DNA target) was obtained for a large collection of 262 serotype Typhi and Paratyphi A strains chosen to be representative of the genetic diversity of these pathogens. This collection included old and recently isolated strains from around the world and strains from various genetic lineages, as previously demonstrated by haplotyping [19] and confirmed [24] or currently in the process of being confirmed by whole-genome sequencing (unpublished). The 100% specificity of the assays was also confirmed with 70 other strains, including NTS from 29 serotypes frequently found associated with bloodstream infections [25], and 42 non-*Salmonella* strains. We are therefore very confident that these CRISPR targets are suitable for the specific detection of all populations of serotypes Typhi and Paratyphi A.

The CRISPR-based PCR assays we have developed were validated on cultured bacterial isolates and may facilitate the identification of these serotypes in addition to (gain of time) or as an alternative to serotyping (lack of availability of typing sera, rough or non motile strains) in clinical or public health laboratories. The next step will be to validate a CRISPR-based PCR assay suitable for use on blood samples in the field. For this purpose, in addition to conventional PCR, we have developed an EvaGreen-based real-time PCR assay with a reasonably good sensitivity when applied to purified DNA. As this real-time PCR was initially developed for well-equipped laboratories (blood culture and real-time PCR technology), we used the same primers as designed for the conventional multiplex PCR, generating amplicons of 244 bp for serotype Typhi and 409 bp for serotype Paratyphi A. These sizes are convenient for rapid differential identification by the agarose gel electrophoresis of PCR products, but it will be necessary to decrease the size of the amplified regions for both serotypes to about 70 to 100 bp to optimize the sensitivity of the real-time PCR assay, an essential requirement when dealing with biological samples, particularly blood. This could be done by modifying the forward primers, TY-F and PA-F, to make them bind further downstream, and the reverse primer, PA-R, to make it bind further upstream, within the ParA1 spacer. An internal control amplification target should also be identified and included in the assay. Of course, other factors such as blood volume, the nature of the anticoagulant used and the presence of a pre-enrichment step are important for the optimization of real-time PCR assays of this type. For laboratories not equipped with real-time PCR technology located in countries in which these diseases are endemic, a nested PCR could be designed to improve the detection limits. This CRISPR-based nested PCR could use TY-F and B2 as the first-round primers and B1 and TY-R as the second-round primers for serotype Typhi (Figure 2). For serotype Paratyphi A, PA-F and A2 could be used as the first-round primers and A1 and PA-R could be used as the second-round primers (Figure 3). The A1, A2, B1 and B2 primers are those previously used for amplification of the entire CRISPR loci [15]. All these primers could potentially be combined, for the development of a multiplex nested PCR targeting both serotypes, Typhi and Paratyphi A. It would also be possible to use a recently developed technique: the loop-mediated isothermal amplification (LAMP) of DNA [26]. This rapid and sensitive amplification method makes use of four to six primers, which recognize six to eight regions of the target DNA (here, CRISPR). This method is suitable for use in resource-limited settings, as a water-bath can be used in place of a thermocycler and visual colorimetric detection by staining with a dye, such as SYBR Green I, can replace gel electrophoresis with ethidium bromide staining [27].

In conclusion, whereas most other published PCR protocols use combinations of different markers for each targeted serotype, with a non optimal validation step, we were able to identify a unique target in a conserved region of the *Salmonella* genome for each targeted serotype and we demonstrated that the two targets were highly stable and specific, in tests of a large, representative collection of strains of serotypes Typhi and Paratyphi A. The multiplex conventional and EvaGreen-based real-time PCR assays described here can be used for the rapid confirmation of identification for these two major serotypes and for differentiation between them, using DNA from cultured isolates. These assays, which are now routinely used at the FNRC-ESS, would be easy to implement in a large range of clinical and public health laboratories with access to PCR technology. Finally, we confirmed that CRISPR was an optimal target for the development of a DNA amplification assay for the detection of any strain of interest with a particular spacer content, provided that a culture of this strain is available.

## Supporting Information

Table S1 Characteristics, CRISPR alleles, sequence types, haplotypes of the 338 *Salmonella* strains, isolates and genomes studied. Excel file.
(XLS)
